# Antidepressants and hypertensive disorders in pregnancy: a retrospective cohort analysis

**DOI:** 10.3389/fpsyt.2025.1614577

**Published:** 2025-11-25

**Authors:** Carolyn Breadon, Shalini Arunogiri, Alisa Turbić, Alex Lavale, Ricardo Maldonado, Jayashri Kulkarni

**Affiliations:** 1 Consultation Liaison Psychiatry Service, Monash University, Austin Hospital Melbourne, Melbourne VIC, Australia; 2 Turning Point, Eastern Health, Monash Addiction Research Centre, Eastern Health Clinical School, Faculty of Medicine Nursing and Health Sciences, Monash University, Arunogiri Melbourne VIC, Australia; 3 Department of Psychiatry, The University of Melbourne, Melbourne, VIC, Australia; 4 Monash University School of Medicine and Health Sciences, Melbourne, VIC, Australia; 5 Power Stats Sydney, Sydney, NSW, Australia; 6 HER Centre Australia, Monash University, Melbourne, VIC, Australia

**Keywords:** essential hypertension, hypertension of pregnancy, pre-eclampsia, eclampsia, depression, anxiety, perinatal depression, antidepressant

## Abstract

**Objective:**

This retrospective cohort study investigated relationships between antidepressant use in pregnancy and hypertensive disorders of pregnancy.

**Design/setting/sample:**

Observational cohort study examining births in an outer-metropolitan maternity hospital in Australia between 2008-2022. 75,308 births were examined.

**Methods:**

Logistic regression analysis considering covariates including maternal age, smoking, BMI, depression, anxiety, schizophrenia or bipolar disorder, gestational diabetes, pre-pregnancy diabetes. The antidepressant treatment cohort was compared with two groups: all births at this hospital within this time period, and a more closely matched depressed/anxious cohort not treated with antidepressants in pregnancy. The overall group of women taking antidepressants in pregnancy was also compared with women taking antihypertensive medications in pregnancy.

**Main outcome measures:**

Clinical diagnoses of hypertension, pre-eclampsia or eclampsia recorded in pregnancy, at birth or the immediate postpartum, as well as treatment with antihypertensive medication.

**Results:**

A statistically significant relationship (*p* = 0.001) between antidepressant use in pregnancy and clinically diagnosed hypertension, OR 2.65, CI 1.45-4.81, when compared with the overall birthing cohort. When covariates were added, including BMI, age and gestational diabetes, this relationship lost statistical significance. The relationship was also non-significant when a depressed/anxious cohort was used as the comparator group: OR 1.49 (*p* = 0.24, CI 0.77 – 2.88). A highly statistically significant relationship was found between antenatal antidepressant use and pre-eclampsia, OR 2.90, (*p* < 0.0005, CI 2.1 – 4.0), which retained significance when covariates were added to the regression analysis (OR 2.07, CI 1.45-2.97, *p* < 0.0005). BMI and gestational diabetes were also significant risk factors for pre-eclampsia in this sample. As in other research, depression was also found to be related to pre-eclampsia at a borderline significant level (*p* = 0.086). Considering the co-administration of antidepressants and antihypertensive medications, a strong relationship was found: OR 2.90, *p* < 0.000, CI 2.13-3.94, aOR 2.02 *p* < 0.000, CI 1.39-2.93. When women taking antidepressants were compared with depressed/anxious peers a similarly significant relationship between antidepressant use and hypertension of pregnancy was found: OR 2.56, (*p* < 0.0005, CI 1.7 – 3.7). We found a highly significant relationship between antidepressant use and eclampsia, OR 2.84 (*p* < 0.0005, CI 2.06 – 3.92), unchanged when compared with the depressed/anxious cohort: OR 2.84 (*p* < 0.0005, CI 2.06 – 3.92).

**Conclusions:**

This study supports existing research suggesting a strong relationship between antidepressant use in pregnancy and hypertensive disorders. Comparison with a depressed/anxious cohort reduces the risk that these underlying conditions could contribute to this finding.

## 1 Introduction

In recent years there has been increasing interest in the relationship between antidepressant use and development of hypertension in pregnancy, with related elevated risk for pre-eclampsia and eclampsia (1, 2). These latter conditions confer increased risk of harm including death to both mother and baby, so reducing rates of all conditions in pregnancy is a clinical priority. Recent studies with varying designs have yielded inconsistent results when examining this relationship (3).

The theoretical underpinning of this relationship relates to the effect of elevated circulating serotonin on peripheral vasculature (4) and on nitric oxide (5). Existing studies implicate not only SSRI (6) and SNRI antidepressants (7) but also tricyclic antidepressants (8), suggesting that these effects are not class-specific.

Gestational hypertension is often defined as new onset hypertension after the 20^th^ week of pregnancy (9). The rate of diagnosis of gestational hypertension in Australia was found to be 3.2% in 2021 and has remained stable at around 3-4% since 2014 (10). Consistent with international data, in 2021 Australian women aged over 40 were increasingly vulnerable to both gestational hypertension (4%) and pre-existing hypertension (2.5%) (11). In addition to advancing age (12), other modifiers to risk of gestational hypertension and related hypertensive disorders of pregnancy appear to be obesity (13), pre-existing diabetes (14, 15), gestational diabetes (16) (hypothesized by some authors to have a common pre-conception causal pathway (17, 18), smoking (19), poverty (20) and ethnicity (21). Interestingly, mental disorders including schizophrenia (22), anxiety (23) and depression (24) have also been shown to have robust associations with hypertensive disorders of pregnancy.

There is some controversy over the relationship between antidepressant use in pregnancy and risk of hypertension. Existing research has been criticized for various methodological flaws which could artificially increase the observed rates of hypertensive disorders in women taking antidepressants (1). Some authors have considered, by contrast, that the use of antidepressants in pregnancy could protect against development of pre-eclampsia and eclampsia due to their role in modifying serotonin availability in the peripheral vasculature (2). However, most existing research has suggested an association between treatment with antidepressant medication and increased risk of gestational hypertension and related disorders. Criticism of these studies has included confounding by indication or severity (25), as well as exclusion of relevant data which has been shown to modify risk including BMI (26), pre-existing (15) or gestational diabetes (27), co-administration of other psychotropic medications, or demographic information such as socioeconomic status, age, and smoking (1). Given this context, this study aimed to investigate the relationship between antidepressant use in pregnancy and hypertensive disorders of pregnancy including information about these comorbid illnesses, coadministration of medications, and lifestyle factors.

## 2 Method

### 2.1 Study design and setting

This was a retrospective review of observational data obtained from clinical records of women receiving antenatal and birthing care at a major, publicly funded outer-metropolitan health service in Australia. This was a clinical database used for the purpose of patient care. Clinicians, including midwives, obstetricians, nurses and other specialist doctors entered clinical and demographic details at antenatal visits and at birth, including postpartum care of mother and baby. Hence diagnosis for relevant conditions, including depression and anxiety, were elicited from patient self-report or from clinician assessment, and not made by specialist psychiatrists or through the use of structured instruments.

### 2.2 Population inclusion and exclusion criteria

All births, including stillbirths^1^, between January 2008-December 2022 were included in this data set for analysis (75,308 births over 15 years of birthing data). The demographic profile of this dataset is examined in detail in a separate paper, however it is useful to note that all patients were drawn from a publicly funded maternity service in a relatively socioeconomically disadvantaged metropolitan area, with a high rate of unemployment, a large percentage of culturally and linguistically diverse populations with a high rate of first generation migration from African, South and South-east Asian nations, and relatively high rates of smoking when compared with overall Australian rates over time.

### 2.3 Ethics approval

Ethics approval for this project, QA 2017.80, was obtained from Western Health Ethics and from Monash University, which oversaw this project since the first approval was granted in 2019. Approval for extension to the examined dataset was provided in 2022.

### 2.4 Statistical analysis

Relevant items from the dataset including year of birth, mother’s age and medical history, current antenatal complications, medications prescribed, illicit drug and alcohol use, and outcomes at birth for both mother and baby were included in data extraction. Total births included in the data extract were 78,482. This dataset was trimmed of all information likely to have been entered inaccurately (e.g. BMI <15, >60). The trimmed data set included 75,308 births. Within this data set 53,670 individual women had sequential or multiple births recorded within the time period specified. Therefore, statistical analysis was adjusted for repeated measures.

The trimmed dataset was transformed using R (28). Statistical analysis was performed using STATA Version 17 (29). Initially, binary logistic regression was performed to discover the relationship between antidepressant prescribing in pregnancy and hypertensive disorders of pregnancy, including pre-eclampsia/eclampsia. Subsequently, covariates including BMI, pre-pregnancy diabetes, gestational diabetes, smoking, age >=35, a current diagnosis of depression, anxiety in pregnancy, bipolar disorder or other major mental illness (including schizophrenia) in pregnancy, or current treatment with antipsychotics in pregnancy, were added to the regression analysis to consider known confounders and their contribution to this relationship.

We considered another way to evaluate the relationship between antidepressant use and hypertension in pregnancy by examining co-prescription of antidepressants and antihypertensives in pregnancy. We performed an unadjusted and adjusted logistic regression analysis of this relationship, using relevant covariates as described above.

In view of the known, complex relationship between depression, antidepressant use and hypertension of pregnancy described above, a cohort of women with a history of diagnoses of anxiety or depression, or current diagnoses of anxiety or depression, with and without antidepressant medication treatment, was identified within the dataset. This group was then used to compare outcomes between women suffering depressive and anxiety symptoms but not treated with medication, and those suffering these conditions who were treated with an antidepressant. Details of this cohort are provided in the Results section of this paper. Relevant comorbidities in this cohort are listed. HELLP Syndrome was considered in this analysis but not finally included^2^.

We further identified a subgroup of treated women for whom the antidepressant name was identified, and collated these into antidepressant subclasses SSRI, SNRI, TCA and “other”. Due to small numbers (further described in Results) we used chi-square tests for each comparison. We did not make a Bonferroni correction. Due to the lower numbers in this data, we did not perform logistic regression to adjust for important covariates such as BMI, maternal age or diabetes.

## 3 Results

**Table 1:** Descriptive statistics: depressed/anxious cohort.

Covariate	No antidepressant treatment in pregnancy: 6412 births (% of untreated cohort)	Antidepressant treatment in pregnancy: 1147 births (% of treated cohort)	Total: 7,559 births (% of overall cohort)
Age 35+	218 (3.40%)	58 (5.06%)	276 (3.65%)
BMI >=35	1293 (20.17%)	294 (25.63%)	1587 (20.99%)
BMI >=30	2299 (35.85%)	500 (43.59%)	2799 (37.03%)
Type II Diabetes diagnosed prior to pregnancy	21 (0.33%)	4 (0.35%)	25 (0.33%)
Gestational Diabetes	1029 (16.05%)	167 (14.56%)	1196 (15.82%)
Social issues in pregnancy*	254 (3.96%)	60 (5.23%)	314 (4.15%)
Poor/no attendance at antenatal appointments	101 (1.58%)	20 (1.74%)	121 (1.6%)
Current diagnosis Bipolar Disorder	94 (1.47%)	45 (3.92%)	139 (1.84%)
Current diagnosis Schizophrenia	19 (0.30%)	16 (1.39%)	35 (0.46%)
Current diagnosis Depression	1826 (28.48%)	712 (62.07%)	2538 (33.58%)
Current diagnosis Anxiety	2017 (31.46%)	416 (36.27%)	2433 (32.19%)
Antipsychotic use in pregnancy	18 (0.28%)	17 (1.48%)	35 (0.46%)
Benzodiazepine use in pregnancy	12 (0.19%)	3 (0.26%)	15 (0.20%)
Sedative use in pregnancy	5 (0.08%)	3 (0.26%)	8 (0.11%)
Anticonvulsant use in pregnancy	15 (0.23%)	4 (0.35%)	19 (0.25%)
Alcohol use in pregnancy	79 (1.23%)	31 (2.70%)	110 (1.46%)
Tobacco smoking in pregnancy	1273 (19.85%)	269 (23.45%)	1542 (20.40%)
Amphetamine use in pregnancy	16 (0.25%)	4 (0.35%)	20 (0.26%)
Cannabis use in pregnancy	193 (3.01%)	39 (3.40%)	232 (3.07%)
Opioid use in pregnancy	35 (0.55%)	6 (0.52%)	41 (0.54%)
Methadone/buprenorphine treatment in pregnancy	69 (1.08%)	19 (1.66%)	88 (1.16%)
Total	6412 (100%)	1147 (100%)	7559 (100%)

*“social issues” in this dataset primarily included homelessness or risk of homelessness, family violence, Child Protection involvement with the family, or imprisonment of one or both parents during the pregnancy period.

### 3.1 Total sample comparison: antidepressant use and hypertension in pregnancy

This binary logistic regression compared women treated with antidepressants with all other women not taking antidepressants in pregnancy. This analysis found a statistically significant relationship between antenatal antidepressant use and essential hypertension. Unadjusted odds ratio suggested that a pregnant woman taking antidepressants was 2.65 times more likely to be diagnosed with hypertension (*p* = 0.001, CI 1.45 – 4.81). Similarly, there was a highly statistically significant relationship between antenatal antidepressant use and pre-eclampsia (OR 2.9, *p* < 0.0005, CI 2.1 – 4.0) and eclampsia (OR 2.84, *p* < 0.0005, CI 2.06 – 3.92).

To explore these relationships further, the following covariates were added to the univariate model: BMI, pre-pregnancy diabetes, gestational diabetes, smoking, age >=35, a current diagnosis of depression, anxiety in pregnancy, bipolar disorder or other major mental illness (including schizophrenia) in pregnancy, or current treatment with antipsychotics in pregnancy.

The adjusted model found somewhat higher rates of hypertension in the context of antidepressant use in pregnancy (OR 1.67), but this relationship was non-significant (*p* = 0.159, CI 0.82-3.40). Other covariates such as BMI (BMI >= 30 OR 4.70, *p* < 0.000, CI 3.670-6.014), gestational diabetes (OR 2.29, *p* < 0.0005, CI 1.79-2.91), and advanced maternal age (age >=35 OR 3.080, p<0.000, CI 2.13-4.44) were strongly associated with hypertension in pregnancy, with statistical significance. Interestingly, tobacco use was negatively associated with hypertension in pregnancy, except for women with BMI>=30.

#### 3.1.1 Pre-eclampsia/eclampsia

When adjusted for the variables listed above, the aOR for women taking antidepressants in pregnancy also suffering pre-eclampsia remained high at 2.07, and statistically significant, with *p* < 0.0005, CI 1.45-2.97. Other significant risk factors found were BMI (BMI>=30, OR 2.05, *p* < 0.0005, CI 1.79-2.35) and gestational diabetes (OR 1.32, *p* = 0.001, CI 1.12-1.55). Notably, the condition of depression in pregnancy also appeared to be related to a higher risk of pre-eclampsia, albeit in a marginally significant way: OR 1.36, *p* = 0.036, CI 1.02-1.82.

Please see **Table 2** for all outcomes listed above, including OR, p value, and CI.

**Table 2:** Comparison of outcomes (hypertension in pregnancy, pre-eclampsia, eclampsia) for total birthing cohort and depressed/anxious cohort).

Cohort examined	Outcome	No antidepressant exposure in pregnancy	Antidepressant exposure in pregnancy	OR	*p>*	CI 95%
Total birthing cohort*	No hypertension in pregnancy	73,841	1,134			
	%	99.57	98.87			
	Hypertension in pregnancy	320	13	**2.65**	**0.001**	**1.45-4.81**
	%	0.43	1.13			
				aOR 1.67	0.159	0.82-3.40
Depressed/anxious cohort**	No hypertension in pregnancy	6,363	1,134			
	%	99.24	98.87			
	Hypertension in pregnancy	49	13	1.49	0.238	0.77-2.88
	%	0.76	1.13			
Total birthing cohort	Anti-hypertensive medication prescribed in pregnancy			**2.90**	**<0.0005**	**2.13-3.94**
				**aOR 2.02**	**<0.0005**	**1.39-2.93**
Total birthing cohort*	No pre-eclampsia in pregnancy	73,201	1,105			
	%	98.71	96.34			
	Pre-eclampsia in pregnancy	960	42	**2.898**	**<0.0005**	**2.10-4.00**
	%	1.29%	3.66			
				**aOR 2.07**	**<0.0005**	**1.45-2.97**
Depressed/anxious cohort**	No pre-eclampsia in pregnancy	6,318	1,105			
		98.53%	96.34%			
	Pre-eclampsia in pregnancy	94	42	**2.555**	**<0.0005**	**1.747-3.735**
	%	1.47%	3.66%			
Total birthing cohort*	No eclampsia	73,182	1,105			
	%	98.68%	96.34%			
	Eclampsia	979	42	**2.841**	**<0.0005**	**2.060-3.919**
	%	1.32%	3.66%			
Depressed/anxious cohort**	No eclampsia	6,318	1,105			
	%	98.53%	96.34%			
	Eclampsia	94	42.00%	**2.841**	**<0.0005**	**2.060-3.919**
	%	1.47%	3.66%			

*Number of observations 75,308. Standard error adjusted for 53,670 clusters in mother ID.

**Number of observations 7,559. Standard error adjusted for 6,151 clusters in mother ID. Bold type indicates significant results (p=<0.05). Note that numbers in bold indicate statistically significant relationships.

### 3.2 Antidepressant use in pregnancy and co-administration of antihypertensive medication

We subsequently examined the relationship between antidepressant use and hypertension in pregnancy. This analysis also utilized the overall birthing cohort. Binary regression analysis showed unadjusted OR of 2.90, *p* < 0.0005, CI 2.13-3.94. Adjusting for the covariates previously listed, aOR remained relatively high at 2.02, and statistically significant at *p* < 0.0005, CI 1.39-2.93. Strong statistically significant relationships were also found with BMI (OR 3.86, *p* < 0.000, CI 3.37-4.41), tobacco use in pregnancy (OR 0.69, *p* = 0.004, CI 0.53-0.89; again a negative association), gestational diabetes (OR 1.72, *p* < 0.000, CI 1.49-1.98) and advanced maternal age (OR 2.36, *p* < 0.000, CI 1.87-2.96).

### 3.3 Anxious/depressed cohort comparison

As mentioned, a cohort of women with a history of anxiety or depression, including postpartum depression, as well as women currently diagnosed with anxiety or depression during this pregnancy, was identified to account in part for unmeasured covariates such as genetic and lifestyle influences on outcomes. The total group of which treatment cohort and comparator group were comprised came to 7,559 births/episodes of care. Of this group, 6,412 babies were not exposed to antidepressants in pregnancy. 1,147 babies were exposed to antidepressants during this pregnancy. Please see **Table 1**: Descriptive statistics for depressed/anxious cohort for a comparison of various factors between the treatment and comparator group in this cohort.

Our results suggest that a pregnant woman taking antidepressants was 1.49 times more likely to be diagnosed with hypertension than her peer who similarly suffered from depression or anxiety. However, this relationship was not statistically significant when adjusted for covariates (aOR 1.49, *p* = 0.24, CI 0.77-2.88). By contrast, the statistically significant relationship found between antenatal antidepressant use and pre-eclampsia found in the previous analysis was maintained when these women were compared with anxious/depressed comparator subjects. The odds ratio suggests that the risk of a pregnant woman taking antidepressants developing pre-eclampsia was 2.56 that for a similarly depressed or anxious woman not taking antidepressants (OR 2.56, *p* < 0.0005, CI 1.75– 3.74). From these findings, it appears that all depressed/anxious women treated with antidepressants who were diagnosed with pre-eclampsia then proceeded to develop eclampsia. The OR for this outcome in relation to antidepressant treatment remained the same as for pre-eclampsia: OR 2.84, *p* < 0.0005, CI 2.06 – 3.92.

### 3.4 SSRI/SNRI/TCA treatment comparison

Our dataset designated antenatal medication treatments in classes, such as “antidepressant”, without further clarification, for 1,119 cases, which we used in the comparison with the larger cohort of women birthing in this setting, as well as comparison with the anxious/depressed cohort. However, there was a further, smaller group of women within this group whose antidepressant medication was further specified. In this group, we found 368 patients treated with SSRIs in pregnancy, 61 patients treated with SNRIs in pregnancy, and 9 patients treated with TCAs. We could not further compare the three specific outcomes we had identified (diagnosis of gestational hypertension; treatment with antihypertensive medications in pregnancy; diagnosis of pre-eclampsia or eclampsia) in relation to the group of women taking TCAs or SNRIs due to low numbers in the comparator set. In this context, we found comparisons of SSRI treatment vs. general population of unadjusted OR 1.70, CI 1.07-2.71, p<0.05 for gestational hypertension, and OR 2.55, CI 1.39-4.42, p<0.01 for pre-eclampsia. There was no significant difference found for either gestational hypertension or pre-eclampsia between the SSRI treated/untreated subgroups with depression and anxiety.

## 4 Discussion

### 4.1 Main findings

This research suggests that the relationship between essential hypertension and antidepressant use in pregnancy is moderated by a number of statistically significant covariates such as tobacco smoking in pregnancy, BMI, gestational diabetes and advanced maternal age, measured through logistic regression and also through the relationship between co-administration of antidepressant and antihypertensive medications. Unmeasured confounders, such as genetic or lifestyle factors related to mental ill-health, also appear to affect the strength of this relationship, such that the use of a comparator group with similar mental health vulnerabilities is likely to provide a helpful comparison for these patients.

Despite the moderation in the strength of the relationship between antidepressant treatment and hypertension of pregnancy when considered in the context of other risk factors, the relationship between antidepressant medication and pre-eclampsia and eclampsia remained strong and statistically significant, even when considered in the context of these other factors.

Notably, patients in this study who suffered depression were more vulnerable to pre-eclampsia, consistent with existing research in this area (24).

Our subgroup analysis of SSRI medications suggests that SSRI treatment for women suffering depression or anxiety in pregnancy does not further elevate rates of gestational hypertension beyond that experienced by the depressed/anxious subgroup.

### 4.2 Strengths

Unlike some larger datasets, our data included information on maternal physical and mental health comorbidities, coadministration of medications, and lifestyle/demographic factors such as BMI, smoking and alcohol and illicit drug use, attendance at antenatal appointments and social issues in pregnancy. These factors are known to have powerful effects on maternal and neonatal outcomes, as demonstrated in this study.

Utilizing a comparison cohort of women with untreated depression or anxiety was helpful in this study, given that several recent reviews and meta-analyses have commented on the methodological flaws inherent in comparing a treatment cohort with a broader population that does not suffer from mental illness (1, 2). Our untreated cohort with a diagnosis of depression or anxiety appeared relatively well-matched to their treated peers on most variables, though notably those treated with antidepressants were more likely to suffer obesity and diabetes (the subject of a separate paper).

A further strength of this research was the use of another measure of hypertensive disease in pregnancy, the administration of antihypertensive medications, a way to identify clinically significant disease and a proxy for severity of illness (30).

### 4.3 Limitations

Given the limitations of the database from which this information was extracted, there are some limitations to this research study. Firstly, diagnoses of depression and anxiety were not made in this dataset by mental health clinicians, or by using a structured instrument, though some of these are themselves subject to claims of bias and misclassification (31). This was a clinical dataset in which patient self-report of mental illness was provided to clinical care providers, primarily midwives and other obstetric staff. This means that some patients may have been misclassified with diagnoses of depression or anxiety who would not have met more stringent clinical criteria for diagnosis. Thus a larger, less unwell comparator group may have been used to compare with women treated with antidepressants than might have been selected if these more stringent criteria had been applied, introducing potential misclassification bias. The use of self-report or non-psychiatric clinician report also meant that mental illness severity or chronicity could not be ascertained, which would have been a useful consideration in further characterizing the treated and untreated cohorts.

A further limitation to this study is the use of patient self-report to delineate patient antidepressant use, whereas there is a known incidence of non-adherence to prescribed medications, especially in pregnancy.

This study attempted to manage residual confounding effects through the use of a depressed/anxious cohort comparison group, as this group may share similar genetic or epigenetic vulnerabilities to adverse health outcomes with those treated with medication for depression or anxiety. However, other sources of residual confounding exist. Given that this group is drawn from a relatively socioeconomically homogeneous (and disadvantaged) group, all of whom birthed at their local, publicly funded maternity service, socioeconomic confounders are probably less likely to impact on the outcomes of this research. This group is also known to be drawn from a highly ethnically and culturally diverse catchment with high rates of migration from Africa, South Asia, and South-East Asia, thus ethnic or cultural factors are less likely to introduce bias into outcomes observed. However, the dataset used to obtain clinical information did not include diet, physical exercise, or stress levels experienced by individual patients, and thus the impact of these factors on outcomes is unknown.

Antidepressant medication class, dose and trimester timing of antidepressant commencement was not available from this dataset, information which has been shown in other studies (32, 33), to have some impact on risk, though not an impact of similar magnitude as metabolic or demographic factors. In particular, SNRI antidepressants have been shown in some studies to have a significant effect on gestational hypertension (7, 34). In a French population-based study between 2004-2019, women taking SNRIs were compared both with unexposed women and with women taking SSRIs in pregnancy, and were found to have significantly greater risk of hypertensive disorders of pregnancy compared with both groups (oddly, with a slightly greater aOR compared with the SSRI cohort, at 2.32 with CI 1.28-4.20 rather than with the unmedicated cohort, at aOR 1.89, CI 1.13-3.18) (35). De Ocampo et al (36) enrolled North American women contacting a drugs exposure hotline into a study involving regular review throughout pregnancy, and found a significantly increased risk of gestational hypertension in women who continued to take SNRIs throughout pregnancy, though these numbers were small (21 women) and 95% CI correspondingly wide (1.33-18.56) indicating substantial uncertainty about this outcome. In this study women who discontinued any antidepressant use or took SSRI antidepressants throughout pregnancy were not found to have a significant increase in risk of gestational hypertension or pre-eclampsia, after adjustment for relevant confounders. However, other studies have not found elevated risk for SNRI antidepressant use (27), and some studies have found elevated risk for SSRI antidepressant use (37–39) while others did not (40, 41).

Trimester of exposure has been found by some authors to have had a significant impact on gestational hypertensive disorders, including Ocampo et al, as cited above, as well as Avalos et al (3), Toh et al (37), and Palmsten et al (40), all of whom found that continuing antidepressant use in the second trimester of pregnancy was associated with increased risk of hypertensive disorders in pregnancy, including eclampsia and pre-eclampsia. Dose of antidepressant has also been found by some authors to have an impact on gestational hypertensive disease; while Yang et al (27) did not find an association between increased risk for hypertensive disease in antidepressant users during pregnancy in their very well-conducted study (aHR 0.89, 95% CI 0.67-1.18) they did note a higher risk for women taking a higher cumulative daily dose (aHR 2.46, 95%CI 1.05-5.74).

### 4.4 Interpretation

Our results support much of the existing literature on the association between antidepressant medication use in pregnancy and increased rates of gestational hypertension, pre-eclampsia and eclampsia. We found that including a relatively well-matched cohort for depression and anxiety weakened this association, suggesting that underlying mental illness also increases the risk for development of hypertension in pregnancy, consistent with existing research (42).

### 4.5 Future directions for research

Further research is required on this issue. It is clear that the pathophysiology of hypertensive disorders in pregnancy, the metabolic influence of obesity and pre-pregnancy insulin resistance on this pathophysiology, as well as the additional metabolic changes of pregnancy, are poorly characterized, though theories about these relationships abound. Similarly, it remains unclear why women with depression or schizophrenia are more vulnerable to gestational hypertensive disease even without active treatment, and why these women when treated have even higher rates of hypertension in pregnancy. Whilst there has been longstanding awareness of cortisol abnormalities, altered inflammatory pathways, and neurohormonal abnormalities in depressed, anxious women, including these women in pregnancy, there is a need to better clarify why, in the context of treatment of depression, these women’s risk of hypertensive disorders remains higher than their untreated peers without mental illness.

Other research has demonstrated the longer-term sequelae for women who suffer gestational hypertensive disease, including later-life cardiovascular disease and diabetes (43–45). In this broader context it becomes even more important to provide effective evidence supporting advice around the care of women who present with the twin challenges of mental ill-health and hypertensive disease in pregnancy.

## 5 Conclusion

Given the ongoing increase in rates of mental ill-health in pregnancy and postpartum and a correlated increase in rates of antidepressant prescribing in pregnancy, as well as increasing rates of interrelated metabolic conditions such as obesity, diabetes and hypertension in pregnancy, this research is a useful contributor to this topic and provides additional information for women and their clinicians in making decisions about medication treatment in pregnancy.

## Data Availability

The raw data supporting the conclusions of this article will be made available by the authors, without undue reservation.

## Footnotes

1 Stillbirth is defined by the World Health Organisation as “a baby who dies after 28 weeks of pregnancy, but before or during birth” (https://www.who.int/health-topics/stillbirth#tab=tab_1).

2 HELLP Syndrome. This life-threatening condition describes a subset of women with eclampsia who suffer HELLP syndrome and is characterized by three hallmark features of haemolysis, elevated liver enzymes and low platelet count. Unfortunately in our dataset the number of women diagnosed with HELLP was too small to be statistically analysed.
